# Supplementary material for: CpG-adjuvanted stable prefusion SARS-CoV-2 spike protein protected hamsters from SARS-CoV-2 challenge
Source: Sci Rep. 2021 Apr 22;11:8761. doi: 10.1038/s41598-021-88283-8 (PMC8062487; doi:10.1038/s41598-021-88283-8)

Supplementary data for:

CpG-adjuvanted stable prefusion SARS-CoV-2 spike protein protected hamsters from SARS-CoV-2 challenge

Chia-En Lien[1], Yi-Jiun Lin[1], Charles Chen[1,2], Wei-Cheng Lian[1], Tsun-Yung Kuo[1,3], John D Campbell[4], Paula Traquina[4], Meei-Yun Lin[1], Luke Tzu-Chi Liu[1], Ya-Shan Chuang[1], Hui-Ying Ko[5], Chun-Che Liao[5], Yen-Hui Chen[5], Jia-Tsrong Jan[6], Hsiu-Hua Ma[6], Cheng-Pu Sun[5], Yin-Shiou Lin[5], Ping-Yi Wu[5], Yu-Chiuan Wang[5], Mi-Hua Tao[5,7*], Yi-Ling Lin[5,7*]

[1]Medigen Vaccine Biologics Corporation, Taipei City, Taiwan

[2]Temple University, Philadelphia, PA 19122, USA

[3]Department of Biotechnology and Animal Science, National Ilan University, Yilan County, Taiwan

[4]Dynavax Technologies, Emeryville, CA 94608, USA

[5]Institute of Biomedical Sciences, Academia Sinica, Taipei, Taiwan

[6]Genomic Research Center, Academia Sinica, Taipei, Taiwan

[7]Biomedical Translation Research Center, Academia Sinica, Taipei, Taiwan

*Corresponding authors: bmtao@ibms.sinica.edu.tw, yll@ibms.sinica.edu.tw

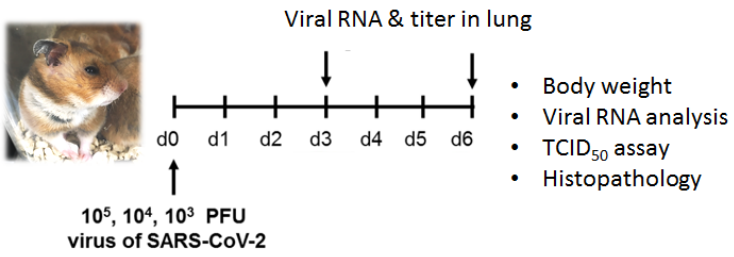

Figure S1. Study design for hamster challenge model development study.

Hamsters were challenged with 10^3, 10^4, or 10^5 PFU of SARS-CoV-2. Body weights were measured each day for up to 6 days after infection and hamsters were euthanized on the third and sixth day after infection for necropsy and tissue sampling.

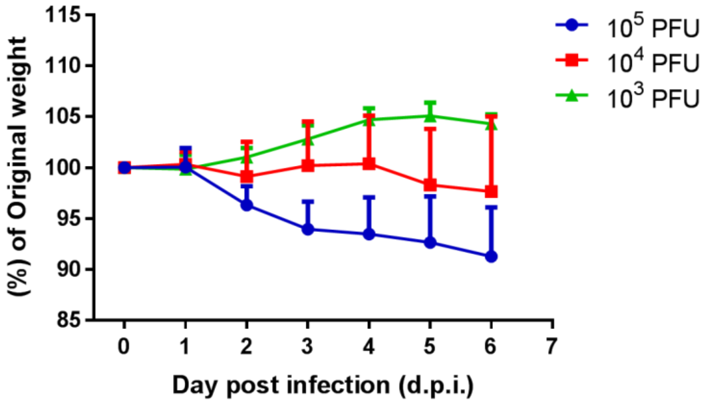

Figure S2. Body weight change in unvaccinated hamsters after the SARS-CoV-2 infection.

Body weights of hamsters were monitored each day after virus challenge for 6 days until euthanization. Results are presented as mean with error bars representing standard error.

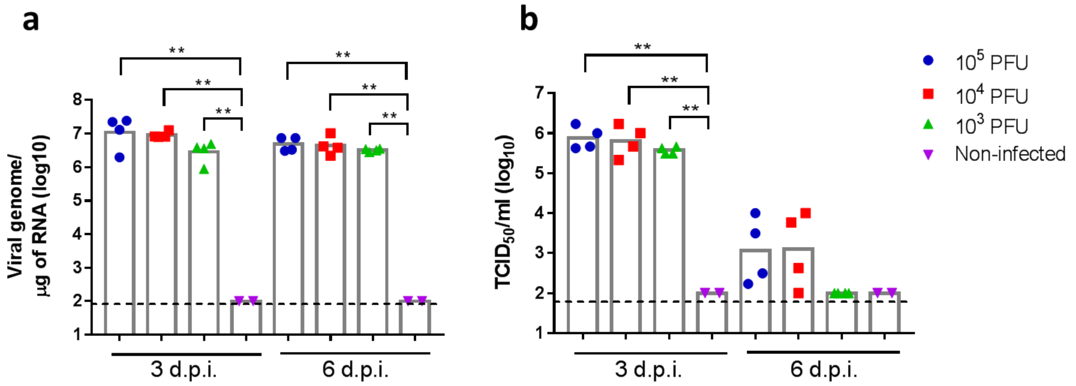

Figure S3. Viral load in unvaccinated hamsters after the SARS-CoV-2 infection.

The hamsters were euthanized at 3 or 6 d.p.i. and lung tissue samples were collected for viral load determination by **a**. quantitative PCR of viral RNA, and **b**. $TCID_{50}$ assay for virus titer. Results are presented as geometric mean with error bars representing 95% confidence interval and statistical significance calculated with Kruskal-Wallis with corrected Dunn's multiple comparisons test. Dotted lines represent lower and limit of detection (100).

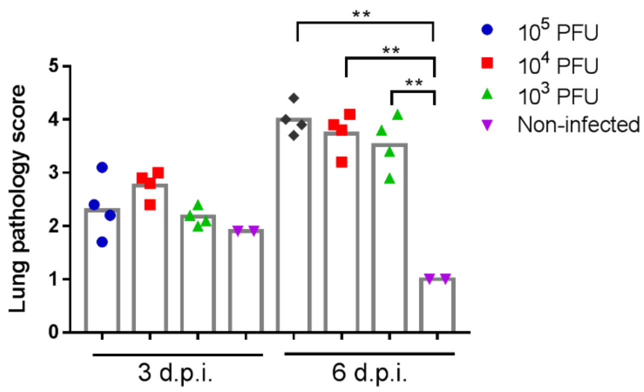

Figure S4. Lung pathology scores in unvaccinated hamsters after the SARS-CoV-2 infection.

The hamsters were euthanized at 3 or 6 d.p.i. and lung tissue samples were collected for sectioning and staining. The histopathology sections were scored as outlined in the methods and the results tabulated. Results are presented as mean of lung pathology scores with error bars representing standard error and statistical significance calculated with Kruskal-Wallis with corrected Dunn's multiple comparisons test between groups.

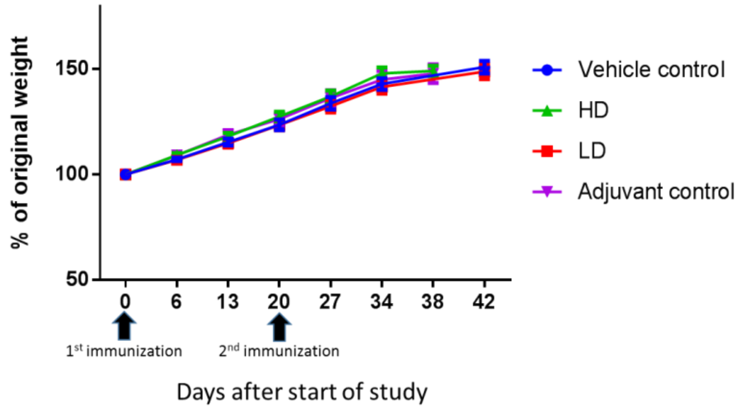

Figure S5. Body weight change in hamsters after start of the study.

Body weights of hamsters were monitored on days 0, 6, 13, 20, 27, 34, 38 (LD and adjuvant only), and 42 (vehicle control and HD) after the first immunization. Results are presented as mean with error bars representing standard error.

Figure S6. Histopathology sections in individual non-infected and infected hamsters in the MVC-COV1901 immunized and control groups.

Hamsters were divided into a non-infected group (n = 8) and 4 infected groups (n = 5 per group) which had received two immunizations at 21 days apart of either vehicle control, adjuvant alone, low dose and high dose of MVC-COV1901. The hamsters were euthanized at 3 or 6 days after SARS-CoV-2 infection and lung tissue samples were collected for sectioning and H&E staining. The representative images of histopathology in lungs from infected and non-infected control group are displayed.

**1/ TA-101, Vehicle control, sacrificed D3, #521** 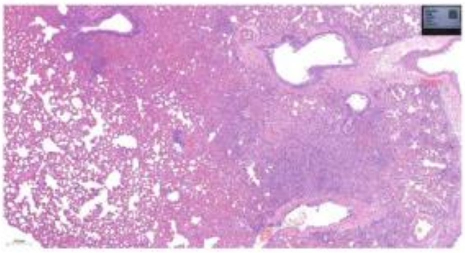	**1/ TA-101, Vehicle control, sacrificed D3, #522** 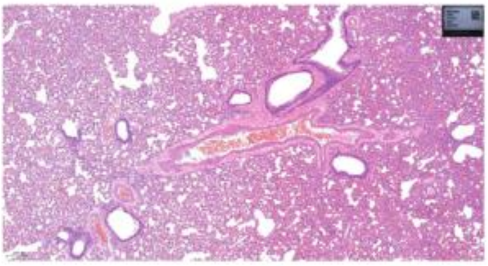
**2/ TA-102, Low dose, sacrificed D3, #526** 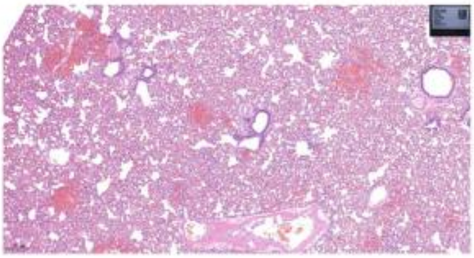	**2/ TA-102, Low dose, sacrificed D3, #527** 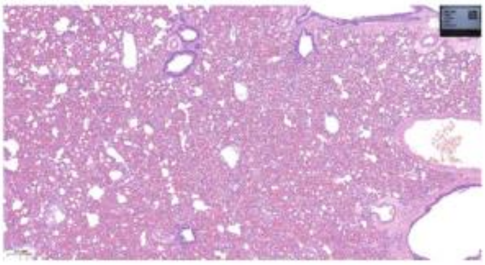
**9/ TA-103, High dose, sacrificed D3, #549** 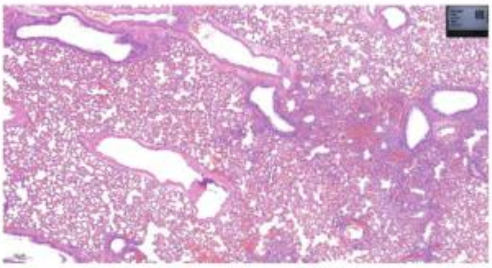	**9/ TA-103, High dose, sacrificed D3, #550** 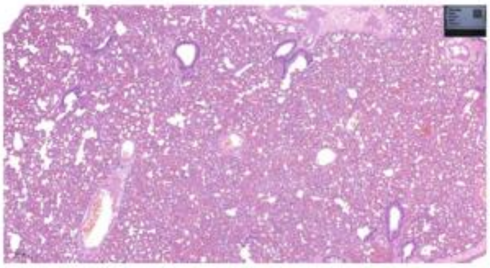
**10/ TA-104, Adjuvant control, sacrificed D3, #554** 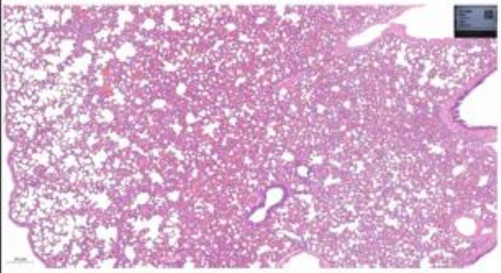	**10/ TA-104, Adjuvant control, sacrificed D3, #555** 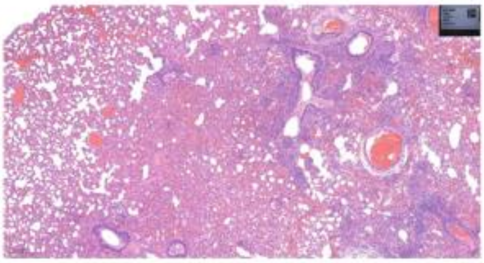

**3/ TA-101, Vehicle control, sacrificed D6, #531**<br>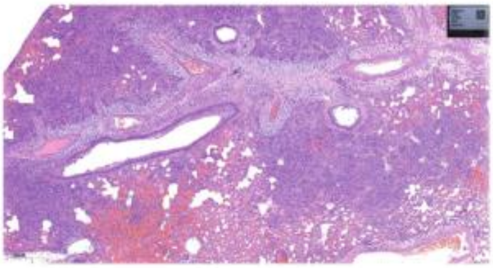	**3/ TA-101, Vehicle control, sacrificed D6, #532**<br>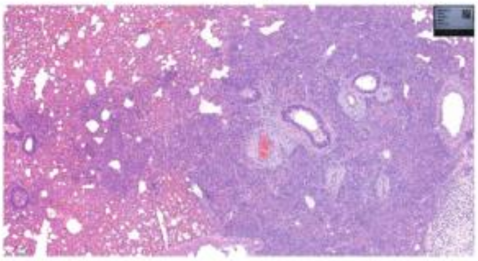
**4/ TA-102, Low dose, sacrificed D6, #536**<br>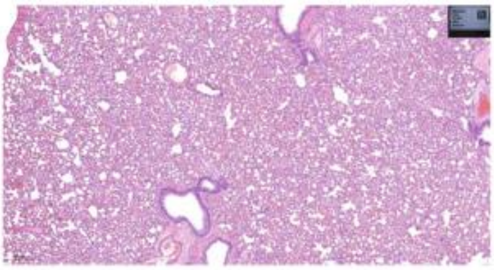	**4/ TA-102, Low dose, sacrificed D6, #537**<br>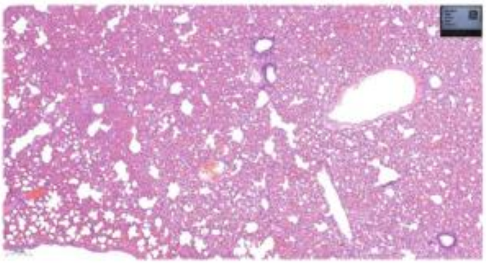
**11/ TA-103, High dose, sacrificed D6, #559**<br>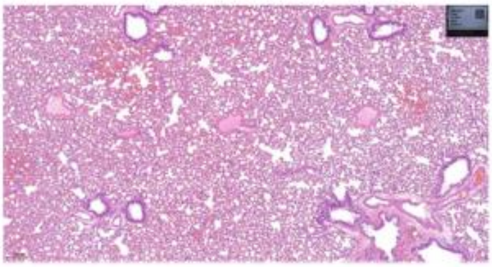	**11/ TA-103, High dose, sacrificed D6, #560**<br>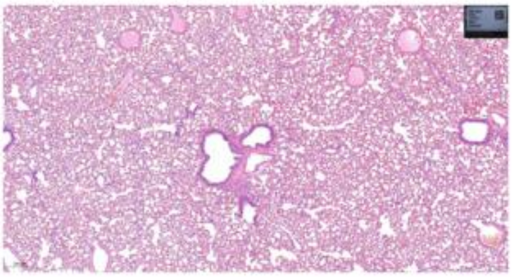
**12/ TA-104, Adjuvant control, sacrificed D6, #564**<br>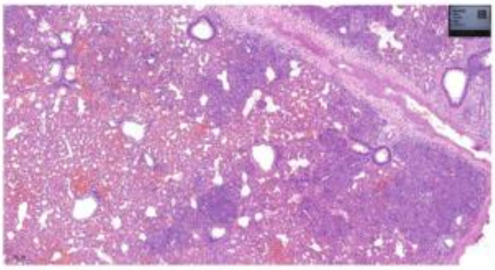	**12/ TA-104, Adjuvant control, sacrificed D6, #565**<br>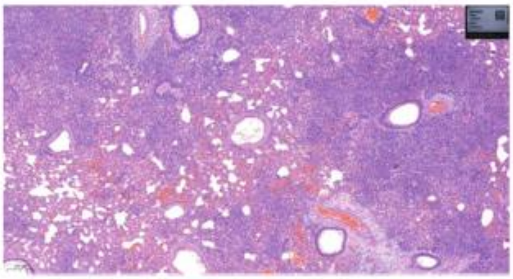

5/ TA-101, non-infected

sacrificed D3, #541

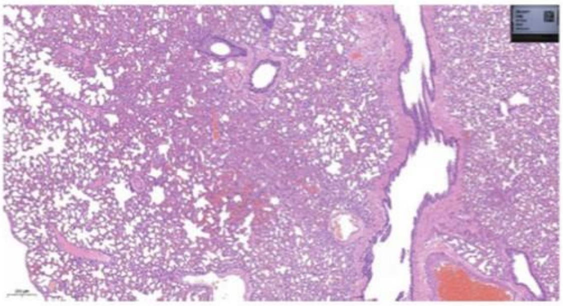

5/ TA-101, non-infected

sacrificed D3, #542

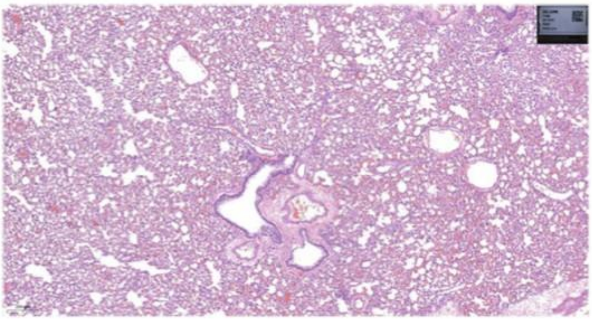

8/ TA-103, non-infected

sacrificed D6, #547

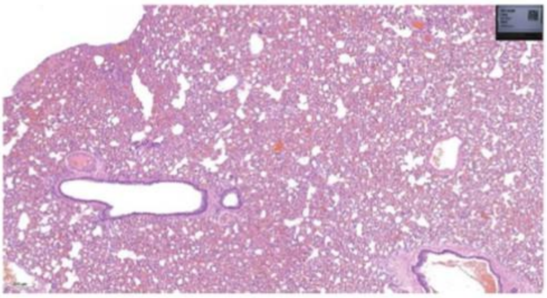

8/ TA-103, non-infected

sacrificed D6, #548

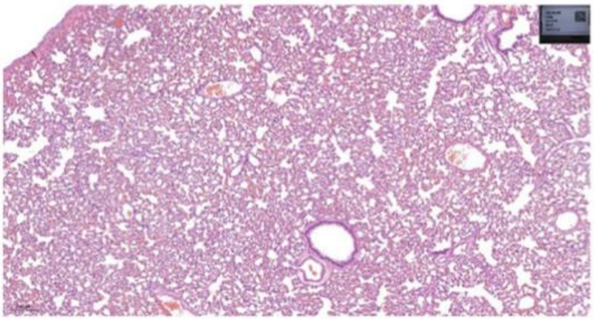

Supplement: Supplementary file 1 — Supplementary Information [file 41598_2021_88283_MOESM1_ESM.pdf]
